# Brain Ageing in Social Anxiety Disorder: An ENIGMA-Anxiety Mega-Analysis Across 26 International Cohorts

**DOI:** 10.64898/2026.07.02.26357108

**Published:** 2026-07-06

**Authors:** Kimberly V. Blake, Jonathan C. Ipser, Alyssa R. Amod, Tobias Kaufmann, Yair Bar-Haim, Jochen Bauer, Ali Bayram, Katja Beesdo-Baum, Laura Blanco-Hinojo, Tiana Borgers, Robin Bülow, Marta Cano, Narcís Cardoner, Christopher R. K. Ching, Soo-Hee Choi, Udo Dannlowski, Christopher G. Davey, Alexander G. G. Doruyter, Kira Flinkenflügel, Gregory A. Fonzo, Tomas Furmark, Dominik Grotegerd, Hans J. Grabe, Tim Hahn, Ben J. Harrison, Alexandre Heeren, Kevin Hilbert, Yoshiyuki Hirano, Joy Hirsch, David Hofmann, Yuko Isobe, Neda Jahanshad, Hamidreza Jamalabadi, Alec J. Jamieson, Andreas Jansen, Jaehyun Edmund Kim, Tilo Kircher, Hitomi Kitagawa, Anna Luisa Klahn, Saskia B. J. Koch, Axel Krug, Harald Kugel, Dasom Lee, Elisabeth J. Leehr, Christine Lochner, Ulrike Lueken, Amirhossein Manzouri, Kristoffer N. T. Månsson, Koji Matsumoto, Susanne Meinert, Alicia Menze, Markus Muehlhan, Benson Mwangi, Igor Nenadić, Ziphozihle Ntwatwa, Hyuntaek Oh, Spiro P. Pantazatos, Martin P. Paulus, Jutta Peterburs, Jesus Pujol, Karin Roelofs, Ramiro Salas, Franklin R. Schneier, Elisabeth Schrammen, Eiji Shimizu, Lisa Sindermann, Theresa M. Slump, Jair C. Soares, Benjamin Straube, Thomas Straube, Murray B. Stein, Ardesheer Talati, Florian Thomas-Odenthal, Sophia I. Thomopoulos, Raşit Tükel, Anna Tyborowska, Marie-José Van Tol, Dick J. Veltman, Roman A. Vogler, Inge Volman, Henry Völzke, P. Michiel Westenberg, Katharina Wittfeld, Mon-Ju Wu, Noga Yair, Tokiko Yoshida, Chen Zhang, Xi Zhu, Giovana B. Zunta-Soares, Peter Zwanzger, Daniel S. Pine, Moji Aghajani, Paul M. Thompson, Nic J. A. Van der Wee, Dan J. Stein, Janna Marie Bas-Hoogendam, Nynke A. Groenewold

**Affiliations:** 1 Department of Psychiatry and Mental Health, Neuroscience Institute, University of Cape Town, South Africa; 2 Department of Psychiatry and Psychotherapy, University of Tübingen, Tübingen, Germany; 3 German Center for Mental Health (DZPG), Partner Site Tübingen, Tübingen, Germany; 4 Centre for Precision Psychiatry, Division of Mental Health and Addiction, Institute of Clinical Medicine, University of Oslo, Oslo, Norway; 5 Tel Aviv University, Tel Aviv, Israel; 6 University Clinic for Radiology, University of Münster, Münster, Germany; 7 Istanbul University, Aziz Sancar Institute of Experimental Medicine, Department of Neuroscience, Turkey; 8 Behavioral Epidemiology, Institute of Clinical Psychology and Psychotherapy, TUD - Dresden University of Technology, Germany; 9 MRI Research Unit, Department of Radiology, Hospital del Mar, Barcelona, Spain; 10 Institute for Translational Psychiatry, University of Münster, Münster, Germany; 11 Institute of Diagnostic Radiology and Neuroradiology, University Medicine Greifswald, Greifswald, Germany; 12 Sant Pau Mental Health Research Group, Institut de Recerca Sant Pau (IR SANT PAU), Barcelona, Spain; 13 Imaging Genetics Center, Mark and Mary Stevens Neuroimaging and Informatics Institute, Keck School of Medicine, University of Southern California, CA, USA; 14 Department of Psychiatry, Seoul National University College of Medicine and Institute of Human Behavioral Medicine, SNU-MRC, Seoul, Republic of Korea; 15 Department of Psychiatry, The University of Melbourne, Parkville, Victoria, 3010, Australia; 16 Division of Nuclear Medicine, Faculty of Medicine and Health Sciences, Stellenbosch University, Stellenbosch, South Africa; 17 Department of Psychiatry and Behavioral Sciences, The University of Texas at Austin Dell Medical School, Austin, TX, USA; 18 Department of Psychology, Uppsala University, Uppsala, Sweden; 19 Department of Psychiatry and Psychotherapy, University Medicine Greifswald, Greifswald, Germany; 20 Psychological Science Research Institute, Université Catholique de Louvain, Louvain-la-Neuve, Belgium; 21 Department of Psychology, HMU Health and Medical University Erfurt, Germany; 22 Research Center for Child Mental Development, Chiba University, Chiba, Japan; 23 United Graduate School of Child Development, The University of Osaka, Japan; 24 Center for Cognitive Behavioral Therapy, Chiba University Hospital, Chiba, Japan; 25 Departments of Psychiatry, Comparative Medicine, & Neuroscience, Yale School of Medicine, New Haven, CT, USA; 26 Institute of Medical Psychology and Systems Neuroscience, University of Münster, Münster, Germany; 27 Department of Cognitive Behavioral Physiology, Graduate School of Medicine, Chiba University, Chiba, Japan; 28 Institute for Advanced Academic Research, Chiba University, Chiba, Japan; 29 Department of Psychiatry and Psychotherapy, University of Marburg, Marburg, Germany; 30 Center for Mind, Brain and Behavior (CMBB), Marburg, Germany; 31 Seoul National University, Seoul, Republic of Korea; 32 Department of Psychiatry and Neurochemistry, Institute of Neuroscience and Physiology, Sahlgrenska Academy, University of Gothenburg, Gothenburg, Sweden; 33 Donders Center for Cognitive Neuroimaging (DCCN), Radboud University Nijmegen, Nijmegen, the Netherlands; 34 Department of Psychiatry and Psychotherapy, University Hospital Bonn, Bonn, Germany; 35 Department of Psychiatry, Seoul National University Hospital, Seoul, Republic of Korea; 36 SAMRC Unit on Risk and Resilience in Mental Disorders, Stellenbosch University, Stellenbosch, South Africa; 37 Department of Psychology, Humboldt-Universität zu Berlin, German Center for Mental Health (DZPG), partner site Berlin-Potsdam, Germany; 38 Department of Clinical Neuroscience, Karolinska Institutet, Stockholm, Sweden; 39 Department of Radiology, Chiba University Hospital, Chiba, Japan; 40 Institute for Translational Neuroscience, University of Münster, Münster, Germany; 41 Medical Faculty Münster, University Hospital Münster, Münster, Germany; 42 Department of Psychology, Faculty of Human Sciences, MSH Medical School Hamburg, Hamburg, Germany; 43 ICAN Institute of Cognitive and Affective Neuroscience, MSH Medical School Hamburg, Hamburg, Germany; 44 Department of Psychiatry and Behavioral Sciences, The University of Texas Health Science Center at Houston, Houston, TX, USA; 45 The Menninger Clinic, Houston, TX, USA; 46 Menninger Department of Psychiatry and Behavioral health, Baylor College of Medicine, Houston, TX, USA; 47 Department of Psychiatry, Columbia University Medical Center, New York, NY, USA; 48 New York State Psychiatric Institute, New York, NY, USA; 49 Icahn School of Medicine at Mount Sinai Hospital, New York, NY, USA; 50 Laureate Institute for Brain Research, Tulsa, OK, USA; 51 Institute of Systems Medicine and Faculty of Human Medicine, MSH Medical School Hamburg, Hamburg, Germany; 52 Behavioural Science Institute (BSI), Radboud University Nijmegen, Nijmegen, the Netherlands; 53 Center for Translational research on Inflammatory Diseases, Michael E DeBakey VA Medical Center, Houston, TX, USA; 54 Department of Psychiatry & School of Public Health, University of California, San Diego, La Jolla, CA, USA; 55 Istanbul University, Istanbul Faculty of Medicine, Department of Psychiatry, Istanbul, Turkey; 56 Center for Clinical Neuroscience and Cognition, University Medical Center Groningen, University of Groningen, Groningen, the Netherlands; 57 Department of Psychiatry, Amsterdam University Medical Center, Amsterdam, the Netherlands; 58 National Education Lab for AI, Radboud University Nijmegen, Nijmegen, the Netherlands; 59 Institute for Community Medicine, University Medicine Greifswald, Greifswald, Germany; 60 Department of Developmental and Educational Psychology, Institute of Psychology, Leiden University, Leiden, the Netherlands; 61 Leiden Institute for Brain and Cognition, Leiden, the Netherlands; 62 The University of Texas at Arlington, Arlington, USA; 63 Department of Psychiatry and Psychotherapy, Ludwig-Maximilian University of Munich, Germany; 64 kbo-Inn-Salzach-Hospital, Wasserberg, Germany; 65 Center for Psychiatry, Psychotherapy, Psychosomatic Medicine and Geriatrics; 66 National Institute of Mental Health, Emotion and Development Branch, USA; 67 Institute of Education & Child Studies, Section Forensic Family & Youth Care, Leiden University, Leiden, the Netherlands; 68 Department of Psychiatry and Psychology, Leiden University Medical Center, Leiden, the Netherlands; 69 Department of Psychiatry, Leiden University Medical Center, Leiden, the Netherlands

## Abstract

Social anxiety disorder (SAD) is among the most prevalent anxiety disorders, and it has been associated with signs of advanced biological ageing. Despite this, brain age research on anxiety disorders remains limited. This mega-analysis investigated brain ageing in adults with SAD within the ENIGMA-Anxiety Working Group. Structural MRI scans from 576 participants with SAD and 1 355 non-affected healthy controls (HCs) across 26 international samples were included. Brain age was estimated from 77 cortical and subcortical regions using a publicly available ENIGMA brain age model. The brain-predicted age difference (brain-PAD) was calculated as the difference between brain age and chronological age. Group and subgroup differences (comorbidity, medication) were assessed using linear mixed-effect models. In the full sample, there was no group difference in brain-PAD (diagnosis (SE)=0.70 (0.37) years, *p*=0.061). In a subgroup of participants with SAD with comorbid anxiety disorders (n=184 SAD, n=1 355 HCs), a brain-PAD of +2.39 (0.93) years (Cohen’s *d*=0.23, *p*FDR=0.003) was observed. This brain-PAD became smaller after exclusion of participants with comorbid agoraphobia and specific phobia, suggesting that these disorders may partly drive the advanced brain-PAD. In conclusion, this ENIGMA-Anxiety mega-analysis did not find evidence of advanced brain ageing in the full sample of adult participants with SAD relative to HCs. However, a sub-analysis suggested that SAD with co-occurring phobic disorders, or the phobic disorders themselves, are associated with neurostructural patterns typical of older brains. Future research could utilise transdiagnostic samples with information on age of onset and disorder duration to further clarify this relation.

## Introduction

Social anxiety disorder (SAD) is defined by a marked fear of one or more social situations and the fear of negative evaluation from others (1). It is one of the most common anxiety disorders, with lifetime prevalence ranging from 0.2%–12.1% across countries (2; 3). Cross-national data demonstrate that SAD is an international public health concern due to its high prevalence and debilitating nature (3–4). Further, SAD seldom exists in isolation, frequently presenting with comorbid psychopathology, including additional anxiety and mood disorders (2; 5). As a result, anxiety disorders have been associated with an early mortality or increased risk of disability in older age (6). Indeed, individuals with anxiety disorders may have a greater risk of developing age-related medical conditions, such as cardiovascular conditions (7). Emerging evidence furthermore suggests that structural brain abnormalities in SAD, such as less grey matter (GM), and white matter (WM) abnormalities, may be associated with advanced biological ageing (8; 9). Therefore, investigating brain ageing in participants with SAD may provide critical insight into the neurobiological mechanisms linking anxiety and brain ageing.

In previous work, SAD has been associated with alterations in brain morphology (10–14). The largest multisite, cross-national mega-analysis to date, performed by the ENIGMA-Anxiety (Enhancing NeuroImaging Genetics through Meta-Analysis) Working Group (15) on participants with SAD (n=1 115) and non-affected healthy controls (HCs; n=2 775; 11), demonstrated smaller bilateral putamen volume in participants with SAD. There was also a significant interaction between SAD and age in the left putamen, with adult, but not adolescent, participants with SAD showing smaller bilateral putamen volumes and larger bilateral pallidum volumes compared to HCs (11).

A promising method for investigating advanced biological ageing in SAD involves the application of machine learning (ML) models of brain age. Brain age ML models are typically trained on large datasets, and then applied to the neuroimaging data of an individual, such as structural or functional magnetic resonance imaging (MRI), to make age predictions (16–19). The deviation between estimated brain age and chronological age is the brain-predicted age difference (brain-PAD). A positive brain-PAD indicates that an individual’s brain appears “older” than would be predicted based on their chronological age (19). The brain age method can be used to develop a reference model derived from healthy individuals, and subsequently to identify structural brain ageing patterns that may be associated with disease (20–21).

The brain-PAD has become an increasingly popular tool for investigating risk for atypical brain ageing in psychiatric disorders: this includes psychotic, mood, and anxiety disorders, which have shown higher brain-PAD in comparison to HCs (22-23; 19; 24–27). Moreover, higher brain-PAD seems to be associated with more severe symptomology in mood and psychotic disorders (24; 28–30). Of note, lower brain-PAD has been reported in psychotropic medication-treated compared with untreated participants with major depressive disorder (MDD) and bipolar disorder, which may reflect neuroprotective effects of these agents (24; 31).

Only three studies have investigated brain ageing in adults with anxiety disorders. The first examined participants with anxiety disorders (18–57 years; generalised anxiety disorder, panic disorder, or SAD; n=67) compared to HCs (n=65) and found that when including antidepressant use as a covariate, anxiety patients had a brain-PAD of +2.91 years compared to HCs (24). The second study, conducted within the ENIGMA-Anxiety Working Group, included a large sample of participants with specific phobia (SPH; n=600) compared to HCs (n=1 134), aged 22–75 years, from 17 sites (32) and reported no group difference in brain-PAD between participants with SPH and HCs. However, a robust diagnosis-by-age interaction was identified across analyses, suggesting subtle, advanced brain ageing in younger formally diagnosed participants with SPH. The third study, conducted within the ENIGMA-Anxiety Working Group, investigated brain ageing in both children and adults with generalised anxiety disorder (GAD), finding no group difference between GAD and HCs. However, the study found a positive relationship between symptom severity and brain-PAD (33). As the study by Han et al. (24) was a transdiagnostic study on a single-site clinical sample, and the studies by Blake et al., (32) and Richier et al. (33) investigated brain-PAD in adults diagnosed with SPH, and GAD, respectively, it is apparent that there is a gap in the literature regarding brain ageing in a large sample of adults with SAD.

The first and primary aim of the present study was to compare brain-PAD in adults with SAD versus HCs, in a large, cross-national cohort. We hypothesised that adults with SAD would have a higher brain-PAD than the HC group. Furthermore, secondary analyses investigated differences in brain-PAD in subgroups of SAD compared to HCs, as defined by comorbidity and medication status. The final aim of the study was to test the hypothesise that SAD symptom severity would be positively associated with brain-PAD.

## Methods

### Study Sample

This multi-site, cross-national ENIGMA-Anxiety mega-analysis included an initial sample of adult participants diagnosed with SAD (n=632) and HCs (n=1 667) from 26 study sites, aged 22–68 years. The study sites, from 10 countries, collected demographic and clinical information for each participant as summarised in Table 1a, b. As reported in Groenewold et al. (11), participants with SAD were excluded if they had a lifetime diagnosis of bipolar disorder, schizophrenia spectrum disorder, or autism spectrum disorder. Participants with SAD with other comorbidities, including other anxiety disorders, MDD, and substance use disorder, were retained, however. HCs were excluded if they had a current or lifetime major psychiatric diagnosis or psychotropic medication use at the time of the scan.

The Human Research Ethics Committee (HREC) at the University of Cape Town granted approval for this study (HREC reference: 675/2021). Each study site obtained informed participant consent and approval from ethical and institutional review boards prior to data collection, and consent for data sharing within the ENIGMA-Anxiety Working Group.

### Image Acquisition and Processing

At the original study sites, investigators processed 3D volumetric structural T1-weighted brain MRI scans using FreeSurfer (version 5.1–7.4.1, with 5.3 being the most commonly used version; 34) and generated FreeSurfer output summary statistics and histograms for visual inspection. FreeSurfer output then underwent established ENIGMA quality control (QC) protocols (https://github.com/ENIGMA-git/ENIGMA-FreeSurfer-protocol; 35-36) by the investigators at the original sites and investigators of the current project. FreeSurfer regions were excluded due to segmentation failure, poor quality segmentations due to processing errors or severe global misclassifications, over/underestimations and misclassifications of brain regions, or motion artifacts. Participants with > 10% missing FreeSurfer data were removed from analyses (SAD n=56, HC n=118). In addition, HCs who were part of a previous ENIGMA-MDD brain age model training were excluded (19; n=194), resulting in a final sample of 576 participants with SAD (lifetime n=15, current n=561) and 1 355 HC (supplementary Figure S1: age density plot).

### Brain Age Model

A ridge regression model developed by the ENIGMA-MDD Working Group (19) was used to predict brain age on the merged dataset of the current project. This brain age model was developed by creating normative models of the association between 77 structural brain features and chronological age in the training control group using the Python-based *sklearn* package version 0.23.1 (37). The model estimated brain age by combining FreeSurfer measures (34) from the right and left hemispheres and calculating the mean volumes of lateral ventricles and subcortical volumes, and cortical thickness and surface area based on the Desikan/Killiany atlas (38). The ENIGMA-MDD model was trained separately on control females (n=14 182) and males (n=12 353), age range: 18–75 years (19). Model validation in a test set of HCs had a mean absolute error (MAE) of 6.50 (SD=4.91) years in male HCs and 6.84 (SD=5.32) years in female HCs. The Pearson correlation between brain age and chronological age was r=0.85 for the male model, and 0.83 in the female models (both *p*<.001). The ENIGMA-MDD brain age model was selected because of its comparable sample characteristics and alignment in data processing pipelines.

### Statistical Analyses

The present study followed a mega-analytic framework as evidence suggests its superiority compared to meta-analysis regarding data preservation, statistical power and flexibility in covariate adjustment (39–41). Brain age estimates were obtained on each site’s FreeSurfer data on a local PC with Python (version 3.6.2), following the methods described in Han et al. (19). The brain-PAD was calculated by subtracting each participant’s chronological age from their brain age.

The fit of the ENIGMA-MDD model with the current study data was assessed by calculating the MAE and Pearson correlation coefficients and explained variance (R2) between brain age and chronological age in the whole sample, and separately for diagnostic groups (SAD; HC) and by sex. Model fit statistics were inspected for each research site. The dataset was checked for normality for brain-PAD, MAE, chronological age and cortical thickness (supplementary Figure S2a-S5b). Outliers were identified based as >1.5 × IQR beyond the quartiles, and extreme outliers as >3 × IQR beyond the quartiles.

Using the *nlme* package (version 3.1–162) in RStudio, a linear mixed-effects model (LME) was used for the first primary analysis to test for a potential group difference in brain-PAD. Brain-PAD was included as the outcome variable (unstandardised **β**-values, measured in years) and diagnosis (SAD; HC) as the predictor variable. To mitigate potential age bias, mean-centred chronological age and mean-centred chronological age squared were included as covariates, in addition to sex. Scan site was included in the models as a random intercept. Diagnosis-by-sex, diagnosis-by-chronological-age (linear) and diagnosis-by-chronological-age-squared (quadratic) interaction terms were included in the model, one at a time, to evaluate if the relationship between the brain-PAD, chronological age and sex differed by group. Interaction variables were removed from the model if they were not significant, to retain best model fit. Bayesian information criterion (BIC) and Akaike information criterion (AIC) determined which model had the best fit (40).

Mean chronological age of the SAD and HC groups differed significantly for two scan sites (FOR2107MS SAD = 30.96 years, HC = 42.04 years; SP Muenster SAD = 38.79 years, HC = 34.07 years; Table 1a). Using the *MatchIt* R package (version 4.5.0), we conducted propensity score matching to match groups (SAD/HC) within these sites on age. Nearest neighbour matching was selected as this approach selects the closest eligible control participant for each case (42). We matched two HCs to one SAD participant on age to optimize sample size, and the distance measure used was the difference between the propensity scores of each case and control unit. The primary LME was rerun in this propensity score matched sample.

A sensitivity analysis was conducted in participants with current SAD only compared to HCs. Comorbidity subgroup analyses were conducted, repeating the model in participants with SAD, with and without comorbid MDD, and in participants with SAD, with and without anxiety disorder comorbidities (GAD, SPH, panic disorder (PD), agoraphobia (AG), separation anxiety, and unspecified anxiety disorder). In addition, medication subgroup analyses were conducted comparing the brain-PAD between HCs and unmedicated SAD, and between HCs and medicated SAD. False discovery rate (FDR) correction was applied to these six subgroup analyses, with a significance threshold of *p*<0.05.

Finally, several post-hoc sensitivity analyses were conducted. First, the primary model was rerun after excluding data from three NESDA sites (SAD n=105, HC n=1,298), as these sites appeared to have lower cortical thickness values than other sites. Next, the primary model was rerun in the SAD group with anxiety disorder comorbidities, compared to HCs, this time removing each anxiety disorder comorbidity subgroup (GAD, PD, AG and SPH) from the entire sample, one at a time.

To address the last aim of the study, analyses were run to determine whether an association between brain ageing and SAD symptom severity was present. For these analyses, data from the Liebowitz Social Anxiety Scale (LSAS; 43), measuring symptom severity in relation to specific social situations, and the trait version of the State Trait Anxiety Inventory (STAI-T; 44), measuring trait anxiety, were selected. A mixed-effects, random intercept regression model was conducted in participants with SAD only (no HCs), with brain-PAD as the outcome variable, LSAS score as the predictor variable and mean-centred chronological age, mean-centred chronological age squared, and sex as covariates. The analysis was repeated with STAI-trait score as the predictor variable. All analyses were conducted in R (version 4.2.1).

## Results

### Model Fit

The brain age model fit with this multi-site ENIGMA-Anxiety SAD dataset was comparable to that seen in prior studies (45; 19; supplementary Table S1). Model fit statistics in the present study were as follows: MAE full sample=6.90 (SD=5.32) years, SAD=7.53 (5.81), HC=6.62 (5.07). When weighted by sample age range (MAE divided by sample age range), the weighted MAE was as follows: full sample=0.15; SAD=0.16; HC=0.14. Finally, Pearson correlations between brain age and chronological age were as follows: full sample=0.69, SAD=0.65, HC=0.72 (supplementary Table S2 and S3: model fit per study site).

### First Aim: Brain-PAD in SAD Versus HCs

**Figure 1:**
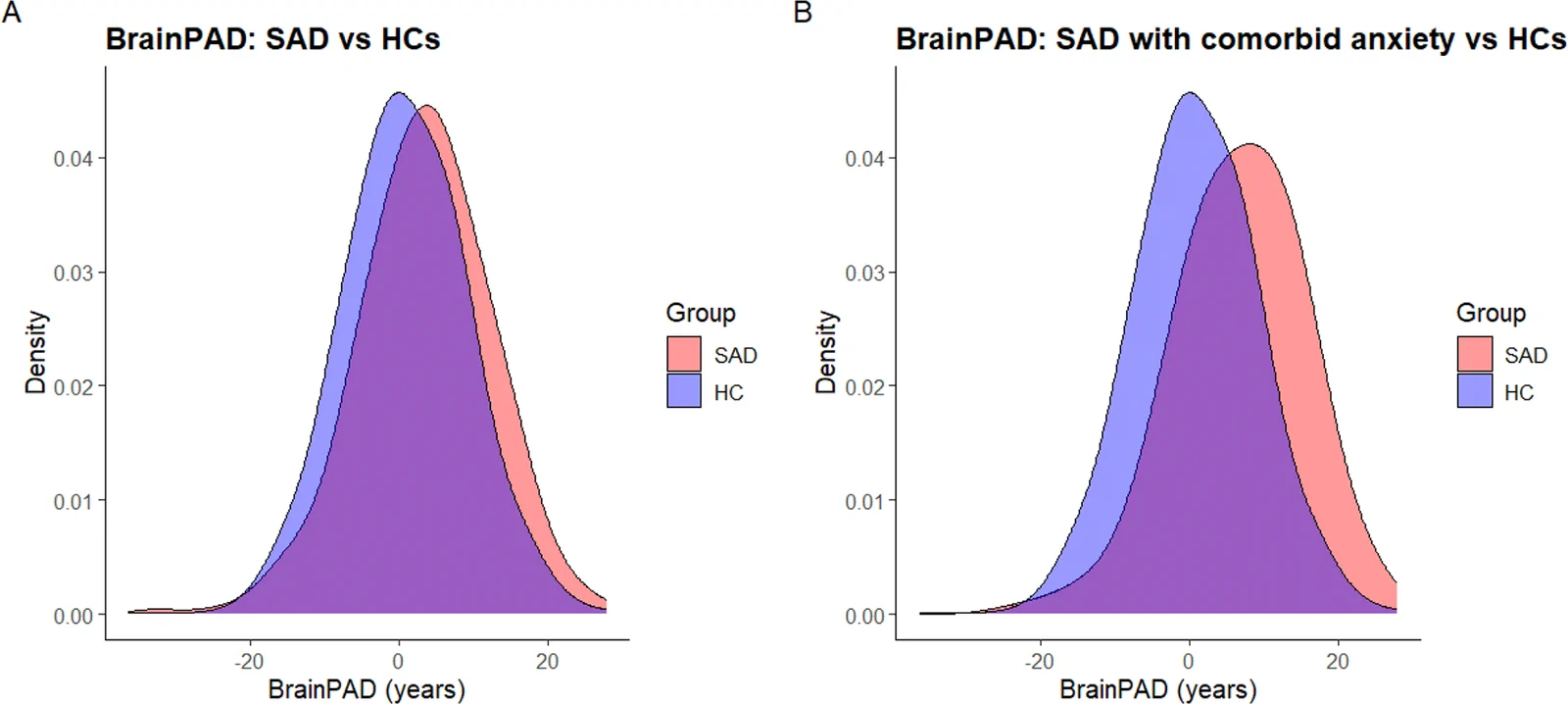
a. Density plot for brain-PAD in SAD and HCs in the full sample. b. Density plot for subgroup of SAD with anxiety disorder comorbidities and HCs. Note. HC, healthy controls; SAD, social anxiety disorder.

Participants with SAD (n=576) had a mean chronological age of 33.25 (SD: ± 9.99), brain age of 36.64 (SD: ± 11.05) years, and mean brain-PAD of 3.39 (SD: ± 8.89) years. HCs (n=1355) had a mean chronological age of 35.00 (SD: ± 11.50), brain age of 35.77 (SD: ± 10.44) years and mean brain-PAD of 0.76 (SD: ± 8.31) years. After adjusting for scanner site, mean-centred chronological age, mean-centred chronological age squared and sex, brain-PAD did not differ significantly between participants with SAD and HCs. There was, however, a trend towards a significant difference (*p*=0.061, Table 2; Cohen’s *d* (95% confidence intervals) = 0.08 (−0.01–0.17), (Figure 1a; supplementary Figure S6: scatterplots per site; supplementary Figure S7: brain-PAD residuals). A significant main effect of mean-centred chronological age and mean-centred chronological age squared was present (Table 2). Data from one participant with SAD and one HC were identified as outliers due to extreme positive and negative brain-PADs in SPSS (version 30, supplementary Table S4, Figure S8). As the outliers did not significantly impact model output, they were retained (supplementary Table S5 for model output).

Interaction terms did not contribute significantly to the model (**β** diagnosis-by-chronological-age=−0.04 (0.03), t-value=−1.08, *p*=0.282; **β** diagnosis-by-chronological-age-squared=−0.01 (0.00), t-value=−1.85, *p*=0.064; **β** diagnosis-by-sex=−0.58 (0.69), t-value=−0.83, *p*=0.404). In certain model combinations where diagnosis-by-chronological-age-squared was included, the group difference in brain-PAD reached significance. However, as the interaction terms did not significantly contribute to the model, they were removed and were treated as supplemental (supplementary Table S6: models including interactions). The model without interaction terms and with the best fit was retained (Table 2), as determined by lower AIC (=1 292.27) and BIC (=1 2981.21).

### Planned Sensitivity Analyses: SAD Versus HCs

The primary LME was rerun in participants with current SAD (removing those with lifetime, but not current, diagnosis; remaining n=561), compared to HCs. The group difference in brain-PAD was similar, as expected, approaching significance (**β**=0.72 (0.38), *p*=0.056, supplementary Table S7 for full model results).

Finally, the model was rerun in the dataset where data from two sites were propensity score matched on chronological age (supplementary Table S8, Figure S9). In this dataset, the brain-PAD was not significantly different between participants with SAD and HCs, and the **β**-value was slightly smaller in size (**β**=0.69(0.39), *p*=0.07; supplementary Table S9).

### Secondary Clinical Subgroup Analyses

Next, we ran the LME in participants with SAD with either current or lifetime comorbid anxiety (PD, GAD, SPH, AG and mixed anxiety-depressive disorder), compared to HCs. A significant brain-PAD of +2.39 (0.93), Cohen’s *d*=0.23, *p*FDR<0.003 was present between the subset of participants with SAD with comorbid anxiety (n=184) compared to HCs (n=1 355, Table 3, Figure 1b; supplementary Table S10, S11 and S12 for subgroup clinical characteristics). However, when we compared participants with SAD with MDD comorbidity to HCs, no group difference in brain-PAD was present (**β**=0.75 (0.49), *p*FDR=0.208, Table 3).

Finally, the LME was run in participants with SAD taking medication at the time of the scan compared to HCs. Here, the diagnosis **β**-value was larger than in the full dataset (**β**=1.18 (0.60)), and the group difference in brain-PAD was borderline significant (*p*=0.051, Table 3), however, this did not survive correction (*p*FDR=0.152). The LMEs run in participants with SAD without medication use, in participants with SAD without anxiety disorder comorbidities, and in participants with SAD without MDD comorbidity (all models: compared to HC) also did not reveal group differences in observed brain-PAD (Table 3).

### Post-Hoc Sensitivity Analyses: Clinical Subgroups

To follow up on the significant group difference in the subset of 184 participants with SAD with comorbid anxiety, we ran multiple sensitivity analyses. A previous ENIGMA study demonstrated that NESDA participants with MDD and HCs have lower average cortical thickness, and therefore overestimated brain age (24). The NESDA study also has high levels of anxiety comorbidity (comorbid MDD and anxiety = 41.4%). Therefore, the LME was repeated after removal of the three NESDA sites. The group difference held (**β**=1.92 (0.70), *p*=0.006, supplementary Table S13 for full model results).

To determine whether any one anxiety disorder comorbidity (GAD, PD, AG, or SPH) was driving the effect seen, a post-hoc sensitivity analysis was run in the SAD group with anxiety comorbidities compared to HCs, removing participants with each comorbidity (GAD, PD, AG and SPH) one diagnosis at a time. All group differences in brain-PAD remained significant, but effect sizes were lower in LMEs after removal of comorbid AG and SPH (supplementary Table S14).

### Second Aim: Symptom Severity

LSAS and STAI-T scores were not significantly associated with brain-PAD in participants with SAD (LSAS score: n=318, **β**LSAS=0.02 (0.02), t-value=0.78, *p*=0.434; STAI-T score: n=248, **β**STAI-T=0.04 (0.04), t-value=0.88, *p*=0.378).

## Discussion

This ENIGMA-Anxiety mega-analysis investigated whether adult participants with SAD showed a larger brain-PAD than HCs. No group difference in brain-PAD was present in the full sample (*p*=0.061) and the effect size was relatively small (Cohen’s *d*=0.08). Secondly, the brain-PAD did not relate to SAD symptom severity, as measured with the LSAS, or trait anxiety, as measured with the STAI-T. Secondary analyses in clinical subgroups did reveal an effect, however, with a positive brain-PAD of +2.39 (0.93) years (Cohen’s *d*=0.23) present in a clinical subgroup of participants with SAD with comorbid anxiety disorders, compared to HCs, after adjusting for chronological age, sex, and study site (*p*FDR=0.003). Post-hoc sensitivity analyses revealed that this group difference was not dependent on a specific comorbid anxiety disorder, as the effect remained significant when data from participants with SAD with specific comorbid anxiety disorders were removed one at a time. In addition, the brain-PAD became smaller after exclusion of participants with comorbid AG and SPH. These findings indicate that SAD with comorbid anxiety disorders may be related to advanced brain ageing, or alternatively, that the comorbid conditions are associated with advanced brain ageing, rather than the SAD itself.

### Brain Ageing in Social Anxiety Disorder

While several studies have reported advanced brain ageing in mood and psychotic disorders (22; 19; 24–25), the present mega-analysis did not identify a group difference in brain-PAD in the full sample of participants with SAD and HCs. Notably, the group difference was close to significance, and when certain interaction variables were included in the model (e.g. diagnosis-by-chronological-age-squared and diagnosis-by-sex; supplementary Table S7), the difference reached significance. However, as these interactions did not significantly contribute to the regression model, they were removed to retain optimal model fit. In a sensitivity analysis including only SAD with current diagnosis (not lifetime) compared to HCs (n=561), the group difference was again just short of significance, with a slightly larger **β**-value than in the full sample (**β**=+0.72 years (0.38), Cohen’s *d*=0.08, *p*=0.056; supplementary Table S13).

An important consideration in brain age research is potential bias related to chronological age. Age is often underestimated in older participants and overestimated in younger participants by brain age models (46). We attempted to manage this bias by rerunning the primary LME in a dataset that was propensity score matched for age between cases and HCs, with the group difference in brain-PAD still not achieving statistical significance. Further, the present study controlled for age by including this variable as a linear and quadratic term in the LMEs (47–48), therefore mitigating age bias.

### Clinical Subgroup Findings

Notably, a positive brain-PAD of +2.39 years was identified in participants with SAD with a comorbid anxiety disorder (GAD, PD, AG, SPH, other), compared to HCs. In contrast, there was no group difference in brain-PAD in participants with SAD without anxiety comorbidities, compared to HCs. The brain-PAD in participants with SAD with comorbid anxiety remained significant in several sensitivity analyses, but when removing participants with phobic disorders (AG and SPH), we observed a decrease in brain-PAD **β**-value by approximately 1 year, indicating that AG and SPH may partially drive the group difference (SAD no AG: **β**=1.72 years (0.72), Cohen’s *d*=0.20, *p*=0.017; SAD no SPH: **β**=1.72 years (0.66), Cohen’s *d*=0.18, *p*=0.009). Sample sizes after removal of comorbid AG and SPH were slightly higher than after removal of comorbid GAD and PD, potentially impacting statistical power (SAD no GAD: **β**=2.88 years (0.76), Cohen’s *d*=0.33, *p*<0.001; SAD no PD: **β**=2.65 years (0.73), Cohen’s *d*=0.32, *p*<0.001; supplementary Table S14). Interestingly, SPH may be considered a marker for subsequent internalising disorders, in part due to its particularly early age of onset during childhood (49–50). These findings underscore the importance of assessing comorbid anxiety presentations in clinical settings, as they may reflect more advanced neurostructural brain ageing.

The size of the present brain-PAD estimate in participants with SAD with anxiety disorder comorbidities (+2.39 years) is smaller than that observed in psychotic disorders (+3–4 years), similar to bipolar disorder (+2 years; 51; 22; 25), and larger than MDD (+1 year; 19). The brain-PAD identified in the present study is comparable in **β**-value and effect size (Cohen’s *d*=0.23) to that found in a cross-disorder sample of participants with anxiety disorders (SAD, GAD, PD) compared to HCs, after correction for antidepressant use (+2.91 years Cohen’s *d*=0.27; 24). The similarities in effect size across studies support the utility of brain-PAD as an index of neurostructural deviations in anxiety disorders.

Multiple biological variables could contribute to advanced brain ageing, such as that observed in the clinical subgroup of participants with SAD with anxiety comorbidities. Oxidative stress may be a major component of anxiety pathology (52), and has been implicated in premature cell death and biological ageing (53–54). In addition, human and animal studies suggest that stress-induced inflammation may harm brain function, in turn, negatively impacting mental health and potentially contributing to the aetiology of anxiety disorders (55).

The positive brain-PAD observed in participants with SAD and anxiety comorbidities may suggest that individuals with multiple anxiety disorders, as opposed to one, exhibit more pervasive impairment (56), although functional impairment was not measured in the present study, and participants with different combinations of comorbidities were not directly compared due to small sample sizes. While we did not see higher levels of symptom severity (LSAS, STAI-T) in the SAD group with anxiety comorbidities (supplementary Table S12) than in other clinical subgroups, these measures may not adequately capture longer term clinical course. Research has shown that patients with multiple anxiety disorders report higher symptom severity than those with one anxiety disorder (57–58), and that long term disability was highest in participants with SAD and multiple anxiety comorbidities, as opposed to GAD only, PD with AG, and PD only (59). Moreover, a study that compared the effect of anxiety disorder combinations in 1 004 outpatients found that level of functioning declined as number of comorbidities increased, and the combination of SAD with PD was the most debilitating (56).

Due to potential subtle neuroprotective effects of psychotropic medication use on brain ageing (51), and because anxiety disorders frequently present with comorbidities (5), participants were further split into clinical subgroups based on medication use and comorbidity with MDD. Contrary to previous brain age literature (24; 31), we found a positive brain-PAD of approximately 1 year in participants with SAD taking psychotropic medication, compared to HCs. However, this difference was not significant after correction for multiple comparisons (*p*FDR = 0.152). Approximately 44% of the clinical subgroup of participants with SAD with anxiety comorbidities took medication at the time of the scan, and close to 77% had comorbid MDD (Supplementary Table S12). However, no group difference in brain-PAD was present in the subgroup of participants with SAD with MDD (50.36% of whom also had comorbid anxiety disorders), compared to HCs, indicating that MDD did not explain the group difference. This could potentially be due to anxiety disorders being the primary diagnoses in our sample, however, this information was not available and therefore could not be formally assessed.

### Social Anxiety Symptom Severity and Trait Anxiety

Our second research aim was to investigate whether there was an association between brain-PAD and symptom severity in participants with SAD. Contrary to previous studies that identified a positive association between the brain-PAD and symptom severity in mood, anxiety and psychotic disorders (24; 28–30), neither metric was associated with brain-PAD in the present study. Another ENIGMA-Anxiety mega-analysis, from which the present sample was derived, also found no associations between subcortical volumes and LSAS and STAI-T score (11). While the LSAS is a well-validated and reliable measure of social anxiety symptoms, its reliance on self-report may lead to under- or overreporting of symptoms (60). In addition, the LSAS is designed to capture current or recent social anxiety severity, as opposed to longer-term severity (43). These measures therefore may not capture additional clinically relevant variables, such as disorder chronicity. Despite our null findings, our results in subgroups of participants with SAD with comorbid anxiety suggest that exploring such clinical characteristics may provide further insight into brain age in anxiety disorders.

### Study Strengths, Limitations and Future Recommendations

This study is the first mega-analysis to investigate brain ageing in a large, cross-national sample of adult participants with SAD. The brain age model showed a moderate fit with the present sample. Further, the mega-analytic design of the present ENIGMA-Anxiety study enabled the use of pre-established preprocessing pipelines and uniform handling of covariates, thereby reducing heterogeneity (61; 41).

Although mega-analyses allow for larger sample sizes, limited power may still be present in this study, particularly for differences of less than one year. Multiple group differences in brain-PAD were close to the significance threshold of *p*=0.05, and a subtle, positive **β**-value of +0.70 years was present in the full sample of participants with SAD. This suggests that an increase in sample size, using a brain age model with lower MAE, or closer harmonisation of neuroimaging data across sites, may have resulted in these group differences reaching significance. An additional limitation of this study is its cross-sectional design, which restricts the ability to infer causal relationships (62; 63). Further, not all sites assessed phobic disorders, therefore it is possible these disorders remained present in the HC group. Finally, the present study was unable to control for certain potentially confounding factors that may impact brain age, such as heavy alcohol consumption and higher blood pressure (64), as data on these lifestyle variables were not consistently available.

Future studies should use more sophisticated brain age models, such as deep learning-based brain age models (65). Further, large-scale transdiagnostic studies with larger clinical subgroup samples may help elucidate how comorbid anxiety disorders, or specific combinations of anxiety disorders, relate to brain ageing.

## Conclusions

The present ENIGMA-Anxiety mega-analysis did not find evidence of advanced brain ageing in the full sample of participants with SAD and HCs. Additionally, no associations between brain-PAD and SAD symptom severity or trait anxiety were present. However, an advanced brain-PAD of +2.39 years was identified in participants with SAD with comorbid anxiety disorders. This effect was smaller following the exclusion of participants with comorbid AG and SPH, indicating that co-occurring phobic disorders may partly drive the advanced brain-PAD observed in this subgroup. Taken together, these findings suggest that SAD comorbid with anxiety disorders, particularly AG and SPH, may reflect greater neurostructural alterations, represented by advanced brain ageing. This implies that these individuals exhibit neurostructural alterations typically observed in older brains. Future work utilising transdiagnostic approaches may benefit from integrating clinical information, such as age of onset and disorder duration, with biological and behavioural data, to further uncover the nuances of this relationship.

## Supplementary Material

Supplement 1

## Figures and Tables

**Table 1a.** Sociodemographic information for each study site included in the main analyses.

Site	Dx interview	Country	All			HC			SAD		
			N	% Female	Mean (SD) age	N	% Female	Mean (SD) age	N	% Female	Mean (SD) age
**BCM/MIND-MB**	SCID	US	101	43.60	31.99 (10.13)	59	47.50	31.32 (9.20)	42	31.10	32.93 (11.35)
**Chiba**	SCID	JP	87	54.00	34.98 (12.26)	72	55.60	35.54 (12.81)	15	46.70	32.27 (9.04)
**Columbia MRT**	Other	US	44	52.30	28.48 (5.81)	18	61.10	29.33 (5.59)	26	46.20	27.88 (5.98)
**Columbia SAD**	Other	US	30	56.70	31.63 (9.59)	15	53.30	32.93 (10.46)	15	60.00	30.33 (8.79)
**Columbia SPP**	SCID	US	34	61.80	33.00 (8.23)	18	44.40*	31.61 (8.18)	16	81.30*	34.56 (8.26)
**DCCN**	MINI	NL	32	53.10	34.88 (10.03)	17	64.70	35.53 (10.64)	15	40.00	34.13 (9.60)
**DelMar**	Other	ES	51	88.20	27.20 (7.25)	27	92.60	27.96 (7.36)	24	83.30	26.33 (7.19(
**Dresden**	CIDI	DE	31	58.10	26.94 (5.43)	19	57.90	26.53 (5.51)	12	58.30	27.58 (5.49)
**FOR2107 MR**	SCID	DE	263	65.40	36.84 (12.44)	239	64.40	37.28 (12.69)	24	75.00	32.50 (8.75)
**FOR2107 MS**	SCID	DE	164	64.60	32.52 (11.64)	141	65.20	30.96 (10.94)**	23	60.90	42.04 (11.50)**
**Houston**	SCID	US	31	45.20	39.81 (9.92)	11	45.50	40.45 (8.34)	20	45.00	39.45 (10.89)
**Istanbul**	SCID	TR	44	36.40	32.41 (6.52)	20	40.00	31.10 (6.04)	24	33.30	33.50 (6.83)
**LFLSAD**	MINI	NL	20	50.00	47.85 (6.56)	10	30.00	50.10 (7.67)	10	70.00	45.60 (4.55)
**UC Louvain**	MINI	BE	29	100.00	24.21 (3.36)	13	100.00	24.54 (4.16)	16	100.00	23.94 (2.67)
**LUMC**	MINI	NL	31	37.50	30.06 (7.51)	17	41.20	28.76 (7.79)	15	33.30	31.53 (7.15)
**MSAD**	SCID	DE	34	32.40	35.56 (11.82)	17	23.50	33.12 (8.49)	17	41.20	38.00 (14.25)
**NESDA Amsterdam**	CIDI	NL	54	57.40	39.02 (9.43)	20	65.00	40.15 (9.26)	34	52.90	38.35 (9.60)
**NESDA Leiden**	CIDI	NL	57	70.20	39.88 (9.14)	26	73.10	41.04 (8.92)	31	67.70	38.90 (9.35)
**NESDA Groningen**	CIDI	NL	44	68.20	39.84 (9.61)	11	54.50	44.64 (9.73)	33	72.70	38.24 (9.17)
**SP Muenster**	SCID	DE	489	59.30	38.22 (11.06)	430	56.50**	38.79 (11.07)**	59	79.70**	34.07 (10.13)**
**TIP**	SCID	DE	20	75.00	27.10 (7.85)	11	90.90	27.82 (10.34)	9	55.60	26.22 (3.35)
**UCSD Sapient Insula**	MINI	US	36	61.10	30.00 (8.15)	20	75.00	30.10 (7.79)	16	43.80	29.88 (8.83)
**Umea Sofie**	SCID	SE	41	78.00	34.12 (9.26)	20	65.00*	34.35 (9.85)	21	90.50*	33.90 (8.90)
**Umea Vox**	MINI & SCID	SE	39	64.10	32.33 (9.13)	20	60.00	34.40 (10.75)	19	68.40	30.16 (6.65)
**Seoul SAD AC**	MINI & SCID	KR	60	43.30	25.67 (2.50)	32	40.60	25.56 (2.49)	28	46.40	25.79 (2.56)
**YSAD**	SCID	AU	64	60.90	23.27 (1.07)	52	63.50	23.37 (1.10)	12	50.00	22.83 (0.84)
**Total across samples**			1931	59.70	34.48 (11.10)	1355	59.40	35.00 (11.50)**	576	60.20	33.25 (9.99)**

Note. Table includes information for the final dataset after exclusions (e.g., due to FreeSurfer quality control failure). CIDI, Composite Interview Diagnostic Instrument; Dx, diagnosis; HC, healthy controls; MINI, Mini-International Neuropsychiatric Interview; N, number; NA, not applicable; SCID, Structured Clinical Interview for DSM-IV disorders; SD, standard deviation;

* *p* < .05;

** *p* < .001 (independent samples T-Test and Chi-square tests comparing SAD and HCs).

**Table 1b.** SAD clinical information for each study site included in the main analyses.

Site	SAD	Comorbidity (lifetime/current)		Medication use		AOO	STAI-T	LSAS	BDI II
	% Current or lifetime	% ANX	% MDD	% any use	% SSRI/SNRI	Mean (SD)	Mean (SD)	Mean (SD)	Mean (SD)
**BCM/MIND-MB**	100 current	45.20 current	2.38/66.70	69.00*	52.40*	NA	NA	NA	NA
**Chiba**	100 current	26.70 current	6.67/20.00*	80.00	53.30*	NA	56.40 (8.18)*	76.80 (30.80)	19.10 (12.50)
**Columbia MRT**	100 current	3.85 current	3.85/19.20	15.40	11.50*	NA	NA	84.40 (17.20)*	NA
**Columbia SAD**	100 current	20.00 current	33.30 lifetime	0.00	0.00	NA	NA	81.40 (16.20)	NA
**Columbia SPP**	100 current	31.20 lifetime	31.20 lifetime	18.80	12.50	10.60 (5.96)	39.10 (9.13)*	NA	NA
**DCCN**	100 current	6.67/13.30	6.67/6.67	0.00	0.00	NA	NA	68.80 (21.70)	13.90 (7.20)
**DelMar**	100 current	0.00/0.00	0.00/0.00	0.00	0.00	NA	43.20 (6.02)*	83.60 (17.30)	NA
**Dresden**	100 current	8.33/25.00	41.70 lifetime	0.00	0.00	12.50 (6.53)*	47.20 (11.50)	52.50 (26.90)	11.20 (6.25)
**FOR2107 MR**	95.83 current, 4.17 lifetime	4.17/41.70	12.50/67.50	83.30	70.80	NA	61.50 (7.55)	NA	23.50 (9.01)
**FOR2107 MS**	78.26 current, 21.74 lifetime	17.40/26.10	21.70/78.30	69.60	69.60	NA	57.70 (10.50)	NA	21.70 (11.50)
**Houston**	100 current	25.00 current	100.00 current	0.00	NA	8.60 (5.50)*	NA	NA	26.70 (15.10)*
**Istanbul**	100 current	8.33 current	0.00/0.00	0.00	0.00	NA	NA	73.80 (18.90)	NA
**LFLSAD**	100 current	10.00/30.00	40.00/10.00	10.00	10.00	6.00 (2.75)	43.80 (9.07)	65.90 (23.00)	13.60 (10.50)
**UC Louvain**	100 current	0.00/0.00	0.00/0.00	0.00	0.00	NA	50.70 (10.20)	77.60 (11.30)	15.40 (8.39)
**LUMC**	100 current	0.00/0.00	13.30 lifetime	13.30	13.30	NA	NA	83.20 (14.60)	19.10 (9.23)
**MSAD**	100 current	29.40 current	17.60/17.60	0.00	0.00	NA	NA	65.70 (11.70)	13.40 (9.84)*
**NESDA Amsterdam**	91.18 current, 8.82 lifetime	5.88/70.60*	23.50/55.90	47.10	35.30	13.00 (6.34)*	NA	NA	NA
**NESDA Leiden**	90.32 current, 9.68 lifetime	25.80/67.70*	35.50/48.40	48.40	35.50	13.60 (9.10)*	NA	NA	NA
**NESDA Groningen**	90.91 current, 9.09 lifetime	18.20/72.70*	36.40/45.40	57.70	39.40	20.40 (12.80)	NA	NA	NA
**SP Muenster**	100 current	8.47/6.78	49.20/1.69	22.00	22.00	17.50 (12.10)*	55.10 (11.10)	60.90 (19.50)*	16.30 (10.60)
**TIP**	100 current	0.00/0.00	44.40/33.30	33.30	33.30	15.90 (7.11)	48.30 (13.60)*	59.90 (16.10)	7.67 (8.08)
**UCSD Sapient Insula**	100 current	0.00/0.00	0.00/00.00	0.00	0.00	NA	50.10 (6.17)*	NA	12.60 (5.10)*
**Umea Sofie**	100 current	0.00/0.00*	0.00/0.00	33.30*	33.30*	16.10 (6.66)*	NA	76.60 (18.80)	NA
**Umea Vox**	100 current	21.00/21.00*	57.90/5.26	0.00	0.00	13.80 (5.31)	46.40 (9.49)	80.20 (17.00)	NA
**Seoul SAD AC**	100 current	3.57/17.90	17.90/10.70*	10.70	10.70	17.20 (5.51)*	53.10 (9.83)*	78.80 (28.20)	17.10 (10.50)
**YSAD**	100 current	58.30 current	8.33/50.00	0.00	0.00	NA	60.50 (8.61)	77.80 (24.60)	NA
**Total across samples**	97.40 current, 2.60 lifetime	6.77/26.40	20.31/28.30*	28.30*	23.09*	15.17 (9.28)*	52.79 (11.31)*	73.88 (21.68)*	17.29 (10.84)*

Note. ANX, anxiety; AOO, age of onset; BDI, Beck Depression Inventory II; LSAS, Liebowitz Social Anxiety Scale; NA, not applicable; SAD, social anxiety disorder; SD, standard deviation; SSRI, selective serotonin reuptake inhibitor; STAI-T, State-Trait Anxiety Inventory – Trait.

* Data not available for all SAD participants within a site.

**Table 2.** Between-group differences (SAD vs HC) in brain-PAD.

	(SE)	t-value	*p*
**Intercept (site)**	2.24 (0.96)	2.33	**0.020**
**Diagnosis**	0.70 (0.37)	1.88	0.061
**Sex**	−0.18 (0.32)	−0.56	0.577
**AgeC**	−0.32 (0.02)	−16.29	**<0.001**
**AgeC^2^**	−4.34 x 10^−3^ (1.47 x 10^−3^)	−2.96	**0.003**

Note. AgeC, mean-centered chronological age; AgeC^2^, mean-centered chronological age squared; HC, healthy controls; SAD, social anxiety disorder; SE, standard error. is measured in years.

**Table 3.** Between group differences in brain-PAD, SAD clinical subgroups compared to HCs

	SAD	HC	Dx				
	n	n	β (SE)	t-value	*p*	*p*FDR	Cohen’s *d* (95% CI)
**SAD (medication use) vs HCs**	163	1 355	1.18(0.60)	1.96	0.051	0.152	0.14(0.04–0.24)
**SAD (ANX comorbidity) vs HCs**	184	1 355	2.39(0.93)	2.58	**<0.001**	**0.003**	0.23(0.13–0.33)
**SAD (MDD comorbidity) vs HCs**	280	1 355	0.75(0.49)	1.55	0.121	0.208	0.08(−0.01–0.18)
**SAD (no medication use) vs HCs**	399	1 355	0.49(0.44)	1.10	0.271	0.326	0.05(−0.04–0.15)
**SAD (no ANX comorbidity) vs HCs**	392	1 355	0.10(0.42)	0.22	0.823	0.823	0.01(−0.08–0.10)
**SAD (no MDD comorbidity) vs HCs**	296	1 355	0.72(0.49)	1.48	0.139	0.208	0.08(−0.02–0.18)

Note. ANX, anxiety; CI, confidence intervals; Dx, diagnosis; FDR, false discovery rate; HC, healthy controls; n, number; SAD, social anxiety disorder; SE, standard error. β is measured in years.
